# Hodgkin’s Lymphoma in the Context of Psoriasis Vulgaris Treated With Immunosuppressive Therapy: A Case Report and Review of Literature

**DOI:** 10.7759/cureus.19490

**Published:** 2021-11-11

**Authors:** Zainab Alali, Bayan H Al Ashour, Munir Alrefaee

**Affiliations:** 1 Internal Medicine/Oncology, Imam Abdulrahman Bin Faisal University, Dammam, SAU; 2 Medicine, Imam Abdulrahman Bin Faisal University, Dammam, SAU; 3 Internal Medicine, King Fahd Hospital of the University, Khobar, SAU

**Keywords:** case report, psoriasis, cd30, cd15, autoimmune, immunosuppressive therapy, hodgkin lymphoma

## Abstract

Psoriasis is a multisystemic chronic inflammatory immune-mediated disorder presenting with multiple clinical manifestations and comorbidities. Studies suggested a significant association between the incidence of malignancy including Hodgkin’s lymphoma and cutaneous T-cell lymphoma and malignancy-related deaths in patients with psoriasis owing to the disease itself, the chronic inflammation, and the immunosuppressive or immunomodulatory effect of drugs. This case report is discussing the condition of an 18-year-old female, who has developed Hodgkin’s lymphoma after receiving immunosuppressive therapy in the context of psoriasis vulgaris flare-up.

## Introduction

Psoriasis has been defined as a chronic inflammatory multisystemic disease, with a prevalence of 0.5-11.4%. As a consequence of the inflammatory nature of the disease, the patient’s clinical manifestations are multisystemic and not only confined to cutaneous tissue [1,2]. Despite the known fact that inflammatory skin plaques are the most common presentation of psoriasis, other presentations and comorbid conditions have been identified. Psychiatric illnesses, obesity, and chronic kidney disease can all be taken as examples [3]. Malignancies, in particular Hodgkin’s lymphoma, have also been suggested to be associated with psoriasis. The National Cancer Institute has estimated the lifetime risk of Hodgkin’s lymphoma to be 0.2%. Moreover, 2.6 per 100,000 people were diagnosed annually, and 0.3 per 100,000 annual deaths occurred. The five-year survival rate in lymphoma patients was noted to be 87.4% [4]. Furthermore, in 2015, local studies have ranked Hodgkin's lymphoma as the 7th and 8th most common malignancy, with an incidence of 3.6% and 2.6% among Saudi males and females, respectively [5]. However, the stated risk appears to be higher in patients with chronic inflammatory disorders who require immunosuppressants throughout the course of their disease [6]. This paper is discussing a case of an 18-year-old female diagnosed with Hodgkin’s lymphoma in the context of active treatment of psoriasis vulgaris with immunosuppressive therapy.

## Case presentation

This report concerns an 18-year-old female, known to have psoriasis vulgaris. The patient initially presented with the typical scaly inflammatory plaques, which were controlled with topical treatment in 2013. Despite her improvement at the time of diagnosis, the patient suffered from a severe relapse in 2018, in which the scaly plaques had involved 80% of her body. As an attempt to treat the flare-up, the patient was initiated on an anti-tumor necrosis factor, etanercept. The patient received the drug on a weekly basis, subcutaneously at a dose of 50 mg, for a period of seven months. Sequentially, the patient started to experience fever, night sweats, cough, dyspnea, and dysphagia. The symptoms were coincident with bilateral progressive supraclavicular swellings, which were increasing in size over a period of four months.

Clinical examination revealed the enlargement of cervical, supraclavicular, and submental nodes. The supraclavicular and cervical lymph nodes were tender upon palpation; however, the submental lymph node was non-tender and described as hard, immobile, with an estimated size of 1.3 cm. Skin examination findings were consistent with active psoriasis in which scaly patches on an erythematous base were reported. Other components of physical examination were insignificant.

**Figure 1:**
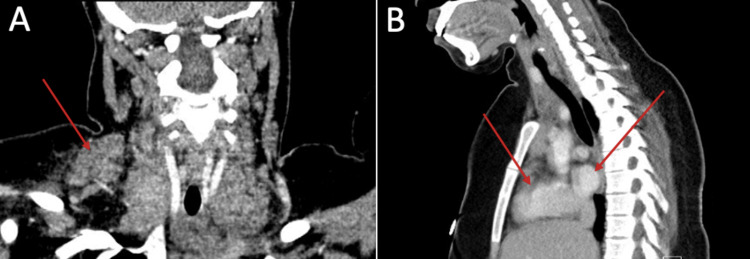
CT scan of an 18-year-old female showing multiple enlarged lymph nodes. A: Coronal cut of the CT scan showing enlarged right supraclavicular lymph node. B: Sagittal cut of the CT scan showing multiple enlarged lymph nodes in the aortopulmonary window and paratracheal region.

The patient was suspected to have tuberculosis; however, the diagnosis was excluded due to the insignificance of the purified protein derivative and sputum culture results. Laboratory investigations revealed high C-reactive protein and high erythrocyte sedimentation rate, and other investigations were normal. The patient’s complaint was further investigated in October 2018 by a computed tomography (CT) scan of the neck and chest (Figure 1). The radiological report was consistent with the patient’s presentation and clinical findings, in which the enlargement of different groups of lymph nodes, at multiple levels, above the diaphragm was recognized. The size of the greatest lymph node was measured to be 4.6 × 3 × 3 cm in the right supraclavicular group. Furthermore, the report stated that the findings of the CT scan are suggestive of lymphoma.

In November 2018, the patient was readmitted for biopsy; however, due to the heavy load on facilities, the biopsy was postponed to the following month. In December 2018, a right cervical lymph node was excised for biopsy under CT guidance. The pathological report denied the presence of malignant cells or granulomas from the provided sample. The report suggested reactive lymphoid hyperplasia and stated that the immunohistochemical markers CD15 and CD30 were absent.

**Figure 2:**
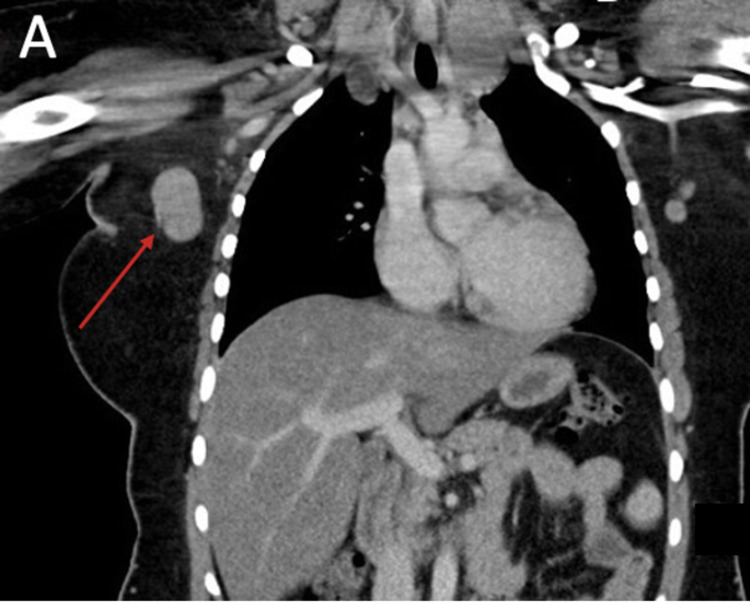
Coronal cut of the CT scan showing enlarged right axillary lymph node.

In February 2019, the patient was readmitted to the medical ward to further investigate her lymphadenopathy. A CT scan showed further progression of the size of lymph nodes (Figure 2). The report stated that the right submental lymph node was estimated to be 1.3 cm in size in the previous scan; however, it had grown to be 1.7 cm and was associated with a necrotic center. Although the lymph nodes in the left aortopulmonary window and right paratracheal region were also enlarged, the growth of the right axillary lymph nodes was of exceptional significance, in which the largest lymph node was estimated to be 2.6 cm in diameter. The scan also presented the mild compression that was applied on the trachea by the enlarged supraclavicular lymph nodes; however, it resumed its normal caliber in the mediastinum. The radiological report has also stated that there were no lung masses, nodules, or pleural effusion. It, however, suggested changes in the density of the liver, which may be indicating fatty liver changes. Thereby, it was recommended that the patient goes under further radiological studies, such as an abdominal and pelvic CT.

**Figure 3:**
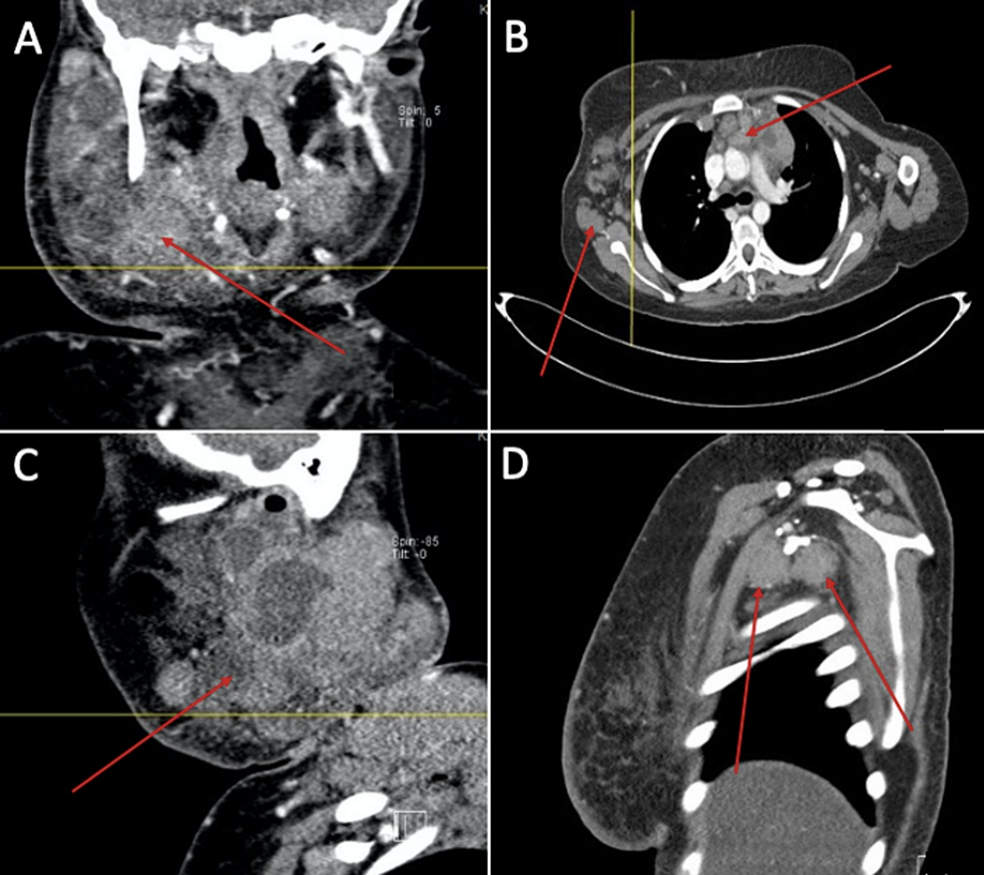
CT scan of an 18-year-old female showing multiple enlarged lymph nodes. A: Neck CT scan, coronal view, showing enlarged right cervical lymph node. B: Chest CT scan, axial view, showing multiple enlarged lymph nodes. C: Neck CT scan, sagittal view, showing enlarged right cervical lymph node. D: Chest CT scan, sagittal view, showing enlarged lymph nodes.

In October 2019, viral causes of lymphadenopathy such as Epstein-Barr virus, cytomegalovirus, and mumps were ruled out. CT scan was repeated and demonstrated further progression of the size of the lymph nodes, in addition to supraglottic narrowing as a result of mass effect (Figure 3). Subsequently, the patient underwent a second excisional biopsy, as she started to experience significant unintentional weight loss, neck enlargement, dysphagia, altered voice, and further enlargement of the lymph nodes. The biopsy was taken from the right axillary lymph node. The pathological report confirmed the diagnosis of nodular sclerosis classical Hodgkin lymphoma stage IIIBSX. Contrary to the previous sample, the immunohistochemical markers CD15 and CD30 were positive. Fortunately, lymphoma was confined to the lymph nodes superior to the diaphragm.

**Figure 4:**
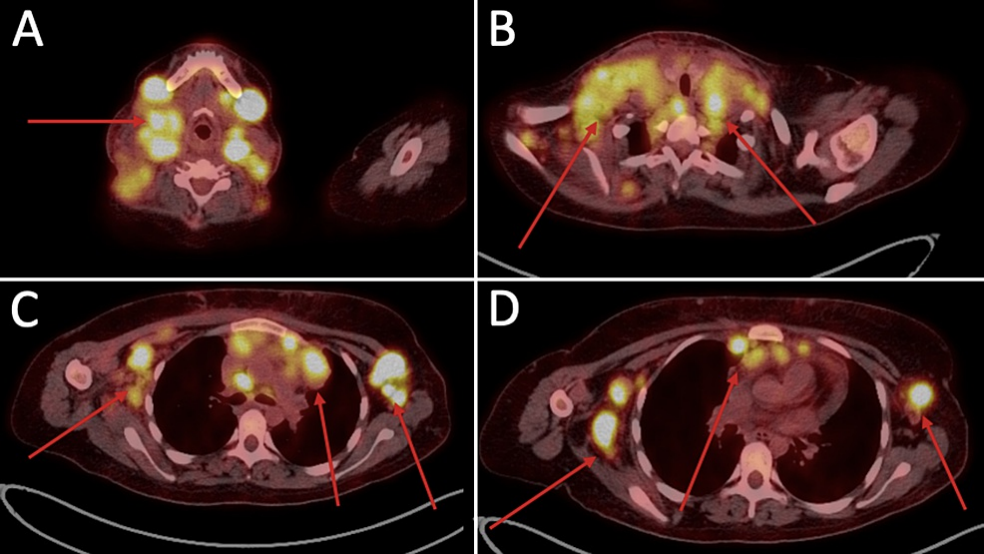
An 18F-FDG PET/CT scan of an 18-year-old female showing lymphadenopathy. A: PET/CT, axial view, representing the bilateral head and neck lymphadenopathy. B: PET/CT, axial view, representing bilateral neck lymphadenopathy. C: PET/CT, axial view, representing axillary and mediastinal lymphadenopathy. D: PET/CT, axial view, representing axillary and mediastinal lymphadenopathy. 18F-FDG PET/CT: positron emission tomography with 2-deoxy-2-[fluorine-18] fluoro-d-glucose integrated with computed tomography; PET/CT: positron emission tomography/computed tomography.

In November 2019, the patient elected to be treated in a tertiary center given her logistics. We were allowed to access and trace her records. The other center ordered a positron emission tomography with 2-deoxy-2-[fluorine-18] fluoro-d-glucose integrated with computed tomography (18F-FDG PET/CT) (Figure 4). The patient’s condition was managed with six cycles of Adriamycin, bleomycin, vinblastine, and dacarbazine (ABVD). Fortunately, the patient underwent complete remission. We had a telephone conversation with the patient, and she expressed the fact of having no active skin lesions as well.

## Discussion

Psoriasis is a prevalent chronic inflammatory disorder affecting 0.5-11.4% of the population worldwide. Recent evidence has suggested that psoriasis is not solely a cutaneous disease, it is in fact a multisystemic chronic inflammatory disorder, presenting with multiple clinical manifestations and comorbidities. Although the classical presentation of a psoriatic patient is inflammatory skin plaques, other comorbidities have been identified. Psoriatic arthritis, autoimmune diseases, chronic kidney disease, non-alcoholic fatty liver disease, obesity, psychiatric disorders, and malignancy are examples of the extracutaneous manifestations associated with psoriasis [3]. The disease was previously recognized as a disease of hyperproliferation, and contrary to such belief, psoriasis is now known to be an immune-mediated disease, in which T lymphocytes, dendritic cells, and cytokines (interleukin-23, interleukin-17, and tumor necrosis factor) were found to be involved in the disorder’s pathogenesis. Due to the wealth of evidence emphasizing the central role of immune dysregulation in psoriasis, therapy has been revolutionized by biological agents that are effective in the short- and long-term outcomes and well-tolerated [1,2]. The biological agents target certain cytokines and immune mediators involved in the pathogenesis of the disease; etanercept, infliximab, and adalimumab are examples of such medication [6]. To our knowledge, the literature is scarce in discussing the long-term outcome and overall mortality of immunosuppressive therapy-related lymphoma in psoriasis patients. A systematic review and meta-analysis of 58 observational studies suggested a significant association between the incidence of malignancy and malignancy-related deaths in patients with psoriasis [7]. The associated malignancies included both hematological and solid tumor malignancies [6]. Furthermore, the mortality risk was noticed to be higher in patients with aggressive disease, and the risk of death was estimated to be 1.22 fold higher in comparison to psoriasis-free populations. The authors proposed such predisposition may be owing to one’s lifestyle, chronic inflammation, or the immunosuppressive/immunomodulatory effect related to psoriasis. A different cohort study using the UK General Practice Research Database suggested that the risk of malignancy due to psoriasis itself is unknown; however, there is a 41% increased risk of cancer-related death in patients receiving treatment for severe psoriasis [8]. An increased incidence of lymphoma, esophageal, liver, and pancreatic cancer was recognized [7]. In particular, the incidence of lymphoma has been shown to be increased in patients with psoriasis, as demonstrated in a large population-based cohort addressing the association with lymphoma only. The excess risk was 7.9/100,000 psoriasis patients per year. In which, the association was particularly stronger with Hodgkin’s lymphoma and cutaneous T-cell lymphoma [9]. Moreover, special considerations are to be made for patients with an ongoing or a history of lymphoproliferative malignancy when treating a psoriatic patient with systemic agents, which result in immunosuppression. Thereby, non-immunosuppressive therapy such as phototherapy and acitretin are recommended in psoriatic patients with such comorbidity as per expert consensus from the United States of America and Europe [9,10]. Although the literature has reported a possible association between psoriasis and certain malignancies, the reported case is only suggestive of such association.

## Conclusions

This case report is discussing the case of an 18-year-old female, who developed Hodgkin’s lymphoma after the treatment of a psoriatic flare-up with etanercept. It highlights two important risk factors for the development of malignancies or other serious comorbidities in patients with psoriasis vulgaris; the inflammatory disease itself and the immunosuppressive medication that can be used throughout the course of the disease. Thereby, emphasis upon the cautious medical prescription of immunosuppressive medication for the treatment of psoriasis is of paramount importance.
